# Epidemiology features of traumatic and non-traumatic spinal cord injury in China, Wuhan

**DOI:** 10.1038/s41598-024-52210-4

**Published:** 2024-01-18

**Authors:** Fater A. Khadour, Younes A. khadour, Ling Meng, Cui XinLi, Tao Xu

**Affiliations:** 1 Department of Rehabilitation, Tongji Hospital, Tongji Medical College, Huazhong University of Science and Technology, 1095#, Jie-Fang Avenue, Qiaokou District, Wuhan, 430030 Hubei China; 2 Department of Rehabilitation, Faculty of Medicine, Al Baath University, Homs, Syria; 3 Department of Physical Therapy, Cairo University, Cairo, Egypt 11835; 4 Department of Physical Therapy, Faculty of Health Science, Al-Baath University, Homs, Syria

**Keywords:** Health care, Risk factors, Medical research, Epidemiology, Neurology, Neurological disorders, Spinal cord diseases

## Abstract

Spinal cord injuries are incredibly disabling and can have fatal consequences. At present, there is a lack of available information regarding the epidemiological characteristics of patients who have experienced spinal cord injury (SCI) in China. This retrospective hospital-based study was conducted in the Rehabilitation department of Wuhan’s Tongji Hospital between 2016 and 2022. A total of 649 individuals diagnosed with SCI (both traumatic and non-traumatic) were admitted during this period. Data regarding various epidemiological features were gathered, including sex, age, etiology, occupation, neurological level of injury, the American Spinal Injury Association Impairment Scale at the time of admission, and information on any accompanying injuries. Out of the 649 cases of SCI, there were 539 cases of traumatic SCI and 110 cases of non-traumatic SCI. The mean age at the time of injury was 45.6 ± 14.8 years. The ratio of male to female patients was higher in traumatic SCI at 2.82:1compared to non-traumatic SCI at 1.68:1. Traffic accidents were the most common cause of TSCI, accounting for (n = 207/539; 38.40%) of cases. On the other hand, neoplasm was the most common cause of NTSCI, accounting for (n = 38/110; 34.54%) of cases. The findings indicated a higher proportion of males, with traffic accidents being the main cause of injury among TSCI patients. It is crucial to prioritize the risk of falling among older adults and allocate more attention to this issue. These results emphasize the need for tailored preventive strategies that consider the unique characteristics of different types of SCI patients.

## Introduction

Spinal cord injury (SCI) is a significant form of damage to the central nervous system, leading to long-lasting physical and psychological consequences. This condition imposes substantial socioeconomic burdens related to healthcare expenses, rehabilitation efforts, and decreased productivity^1–5^. Consequently, it is crucial to make extensive endeavors in predicting and preventing SCI to enhance the overall health and well-being of the population. Educational programs that are developed based on epidemiological research have demonstrated success in reducing the occurrence of traumatic SCI^6–9^.

The National Spinal Cord Injury Statistical Center (NSCISC) in the United States continually monitors epidemiological trends related to age, sex, and the completeness of spinal cord injury (SCI). Recent data from the NSCISC indicates a gradual increase in the prevalence of SCI among older and adult individuals, females, and those with incomplete injuries^10–12^.

Only Japan and Taiwan have established systems for counting and registering traumatic SCI cases from an epidemiological perspective in Asia. Other Asian countries, including China, have not adopted the same approach^13^. In China specifically, individuals with SCI are categorized as having a "physical disability" and are grouped with other types of disabilities. As a result, the exact incidence of SCI in China can only be estimated indirectly^13^. More specific data collection and classification are needed to accurately assess the prevalence and characteristics of SCI in China compared to countries with dedicated SCI registries.

Recent studies conducted in China, including those by Chen et al.^14^, Yuan et al.^15^, and Hu et al.^16^, have examined national demographic data specific to spinal cord injury (SCI). These studies have shed light on the epidemiological characteristics and trends of SCI in the country. Hu et al.^16^ found that falls were the leading cause of traumatic SCI in 1999, accounting for 52.4%. Traffic accidents were the second most common cause, representing 26.4% during that period. However, the study conducted by Yuan et al.^15^ revealed that traffic accidents were the main causes of SCI, followed by falls. These findings indicate a change in the trends and causes of traumatic SCI in China.

Interpreting changes in spinal cord injury (SCI) epidemiology globally involves considering numerous explanations and hypotheses. It is crucial to understand the demographic characteristics and cultural context of each country, as they are integral to interpreting the etiology of SCI. For instance, in Bangladesh, a developing Asian country with a low-income economy and a predominantly rural population, falls contribute to a substantial proportion (63.0%) of SCI cases. These injuries primarily affect individuals aged 10 to 40 years and are often attributed to falls from trees or the act of carrying heavy objects on the head. This highlights the specific risk factors and circumstances that play a role in SCI occurrence within this population. Conversely, in eastern Canada, a developed country with a high-income economy and a predominantly urban population, the average age at the time of SCI is 55.4 years. Falls account for a smaller portion (19.1%) of SCI. Nevertheless, falls emerge as the leading cause of SCI among the elderly population (age ≥ 60 years), constituting 47% of cases^17^.

Unlike many developed countries, China lacks a national spinal cord injury (SCI) registration system^18^. Consequently, existing research on SCI in China is primarily based on hospital data^5,19^. Most of these studies focus on traumatic spinal cord injuries (TSCIs)^20,21^, while others examine epidemiological characteristics and injury features rather than the incidence rate of SCI^20,22^. It is important to note that the epidemiological features of SCI can vary across different geographic locations, making population-level epidemiological studies crucial in understanding the overall picture of SCI in China. Given the absence of a nationwide SCI database, conducting such studies at the population level becomes essential. Wuhan, situated in central China, is recognized as one of the largest cities in Hubei Province. It spans an area of 8494 square kilometers and had an estimated population of approximately 8.6 million residents as of 2022. Wuhan is an important economic center in China. It has a thriving economy with a diverse range of industries, including manufacturing, technology, automotive, pharmaceuticals, and more. In addition, it has witnessed significant urbanization in recent decades. The city has experienced rapid growth and infrastructure development, including expanding transportation networks, residential areas, commercial centers, and public facilities^23^.

Our understanding of the epidemiological characteristics of spinal cord injury (SCI) in Wuhan is currently limited as there is scarce documented information available. Therefore, the objective of this study was to provide epidemiological data on individuals with SCI who received treatment at Tongji Hospital in Wuhan City. This research aims to contribute to informed decision-making regarding the allocation of healthcare resources, with the ultimate goal of reducing the social and financial burdens associated with SCI in Wuhan.

## Methods

To our knowledge, there is no existing nationwide population-based registration system for spinal cord injuries (both traumatic and non-traumatic) in Wuhan. A retrospective review was conducted on the medical records of the patients who were admitted or transferred to the rehabilitation department of Tongji Hospital, spanning from January 1, 2016, to December 31, 2022. Tongji Hospital is a tertiary care hospital that has a rehabilitation unit (inpatient and outpatient) for treating patients with SCI (Traumatic and Non-traumatic injury); this unit is equipped with the latest apparatus and equipment, as well as a professional medical team that uses the best and latest medical techniques to treat patients with SCI.

The International Classification of Disease Version 10 (ICD-10) and the TSCI diagnosis code were utilized during this review. The data included in this study encompassed various factors such as age, gender, cause of injury, occupation, American Spinal Injury Association (ASIA) Impairment Scale (AIS), level of injury, and associated injuries.

The study included specific criteria for participant inclusion. These criteria encompassed both traumatic and non-traumatic spinal cord injuries or cauda equina injuries that occurred in Wuhan, specifically among patients who were hospitalized at Tongji Hospital at the time of their injury. The International Spinal Cord Injury Core Data Set (version 1.1) was utilized for this study. On the other hand, the study had specific criteria for participant exclusion. These criteria involved individuals with intervertebral disc disease, spinal fractures without spinal cord injury, patients with incomplete medical records, medical records that had unclear diagnoses, and individuals who suffered fatal injuries but were never admitted to the hospital.

In the present study, the participants were categorized into six different age groups, consistent with the approach taken in numerous previous studies^24^. These age groups were as follows: 0–19, 20–29, 30–39, 40–49, 50–59, and 60 years and above. Marital status was recorded and classified as married, unmarried, divorced, or widowed.

The etiology of the injuries was classified according to the cause. This classification for traumatic spinal cord injuries (TSCI) included traffic accidents, which were further divided into four-wheeled vehicles, two-wheeled vehicles, bicycles, and pedestrians. Falls were also considered, with low falls defined as falls from a height of less than 1 m and high falls defined as falls from a height of 1 m or more^25^. Other causes of injury included injuries caused by falling objects, machinery-related injuries, sports-related injuries, as well as non-traumatic spinal cord injuries (NTSCI) resulting from conditions such as demyelinating disease, neoplasm, degenerative disease, vascular disease, and infectious diseases. Occupations were classified into various categories, including workers, farmers, government employees, students, retired individuals, and others. The neurological level of injury was determined based on the segment of the spinal cord affected, including cervical, thoracic, lumbar, and sacral segments. The missing data was dropped from the final results of this study and only was presented as a number (%) in Table 1. The research conducted in this study received approval from the Ethics Committee of Tongji Hospital, Tongji Medical College, under the reference number TJ-IRP20230413.

**Table 1:** Characteristics of patients with SCI from 2016 to 2022.

Variable	Traumatic	Non-traumatic	Overall
Age			
0–19	28 (5.19%)	2(1.81%)	30(4.64%)
20–29	95 (17.62%)	3(2.72%)	98(15.17%)
30–39	199(36.92%)	10(9.09%)	209(32.01%)
40–49	103(19.10%)	17(15.45%)	120(18.52%)
50–59	57(10.57%)	27(24.54%)	84(12.94%)
≥ 60	53(9.83%)	50(45.45%)	103(15.87%)
Missing data	4 (0.74%)	1(0.90%)	5 (0.77%)
Mean age at injury	35.4 ± 13.2	54.8 ± 16.4	45.6 ± 14.8
Gender			
Male	398 (73.84%)	69(62.73%)	467(72.02%)
Female	141(26.16%)	41(37.27%)	182(27.98%)
Education level			
Illiterate	17(3.15%)	4(3.63%)	21(3.25%)
Elementary School	101(18.73%)	15(13.63%)	116(17.77%)
Middle school	208(38.21%)	19(16.36%)	227(34.97%)
High School	107(19.85%)	38(34.54%)	142(21.88%)
College or more	108(20.03%)	35(31.81%)	143(22.13%)
Occupation			
Farmer	80(14.84%)	30(27.27%)	110(17.02%)
Worker	167(30.98%)	18(16.36%)	185(28.20%)
Government- offices	86(15.95%)	12(10.90%)	98(15.17%)
Retired	34(6.30%)	36(32.72%)	70(10.83%)
Students	44(8.16%)	3(2.72%)	47(7.27%)
Others*	128 (23.74%)	11(11.0%)	139(21.51%)
Marital status			
Married	357(69.57%)	68(61.81%)	425(65.51%)
Unmarried	119(22.07%)	28(25.45%)	147(22.58%)
Divorced	38(7.05%)	7(6.36%)	45(6.93%)
Widowed	23(4.26%)	5(5.54%)	28(4.31%)
Missing data	2(0.37%)	2(1.81%)	4(0.61%)
Ethic groups			
Han	471(87.38%)	79(71.81%)	550(84.74%)
Zhuang	22(4.08%)	12(10.90%)	34(5.26%)
Miao	23(4.26%)	10(9.09%)	33(5.02%)
Tujia	19(3.52%)	7(6.36%)	26(4.02%)
Missing data	4(0.742%)	2(1.81%)	6(0.92%)
AIS			
A	183(34.87%)	4(3.63%)	187(28.69%)
B	192(35.62%)	17(15.45%)	209(32.35%)
C	114(21.15%)	28(25.54%)	142(21.78%)
D	52(9.64%)	59(53.63%)	111(17.18%)
Type of disability			
Tetraplegia	223(41.37%)	18(16.36%)	241(37.24%)
Paraplegia	316(58.62%)	92(83.63%)	408(62.76%)
Level of injury			
Cervical cord	223(41.30)	49(44.54%)	272(41.91%)
Thoracic cord	168(31.16%)	18(16.36%)	186(28.67%)
Lumbar cord	112(20.77%)	35(31.81%)	147(22.65%)
Sacral cord	36(6.67%)	8(7.27%)	44(6.77%)

Other* included unemployed individuals and self-employed individuals.

Values are presented as number (%) or mean ± standard deviation.

AIS, American Spinal Injury Association impairment scale.

### Data analysis

Statistical analyses were conducted using the SPSS version 23.0 to analyze the data. The data were organized in an Excel spreadsheet for statistical calculations and tabulation. Descriptive statistics were computed to summarize the baseline variables. Patient characteristics were presented as mean values with standard deviations. Frequency analysis was utilized to analyze the data and calculate percentages. The predetermined level of statistical significance was set at p < 0.05.

### Ethical approval

The ethics committee of Tongji Hospital, Tongji Medical College, Huazhong University of Science and Technology Institutional Review Board Consent Letter—IRB TJ-IRP20230413 approved this study. All procedures were conducted under the ethical principles outlined in the 1964 Declaration of Helsinki and its subsequent revisions. All our methods were carried out under relevant guidelines and regulations. Informed consent was obtained from all the participants and their legal guardian(s) (illiterate participants). We explained the purpose of the study to the patients and their family members before using their data in this study. It was all voluntary; no names were taken, so we provided anonymous data collection.

## Results

This study initially considered a total of 697 cases of spinal cord injury (SCI) occurring between 2016 and 2022. However, during the screening process, 48 cases were excluded due to various reasons. These reasons encompassed 8 cases with traumatic brain injury,17 cases with fractures of the spinal column but without accompanying SCI, 4 cases with heart disease, 6 cases with diagnoses that were unclear, and 13 cases with incomplete medical records or uncertain diagnoses. The percentage of missing data was less than 2% for all relevant variables.

### General characteristics of TSCI and NTSCI patients between 2016 and 2022

The demographic characteristics of individuals with spinal cord injury (SCI) are presented in Table 1. A total of 649 cases of spinal cord injury were included in the study, with 539 cases classified as traumatic and 110 cases classified as non-traumatic. Among the traumatic SCI group, 398 cases (73.84%) were males, while 141 cases (26.16%) were females. In the non-traumatic SCI group, 69 cases (62.73%) were males, and 41 cases (37.27%) were females. The proportion of male patients was significantly higher in the traumatic SCI group, with a ratio of 2.82:1, compared to the non-traumatic SCI group, with a ratio of 1.68:1. The mean age of the entire cohort was 45.6 ± 14.8 years, with the traumatic SCI group having a mean age of 35.4 ± 13.2 years and the non-traumatic SCI group having a mean age of 54.8 ± 16.4 years. The highest proportion of patients in the 30–39 years age group in the overall cohort (32.01%) and the majority of patients in the traumatic SCI group (36.92%) were also within the 30–39 years age range. On the other hand, the majority of patients in the non-traumatic SCI group (46.36%) belonged to the ≥ 60 years age range.

Regarding educational attainment, a majority of the participants (52.74%; n = 343) had completed elementary or middle school, while a small proportion (3.25%; n = 21) were classified as illiterate. The most prevalent occupational category among the participants was workers (28.20%; n = 185), followed by other categories such as unemployed individuals and self-employed individuals (23.74%; n = 128) and farmers (17.02%; n = 110). The majority of the patients (65.51%; n = 425) were married, while (22.58%; n = 147) were unmarried. The Han ethnic group constituted the dominant ethnic group, accounting for (84.74%; n = 550) of the total population, while other ethnic groups included Zhuang (5.26%; n = 34), Miao (5.02%; n = 33) and Tujia (4.02%; n = 26), Table 1.

### Etiology

**Figure 1:**
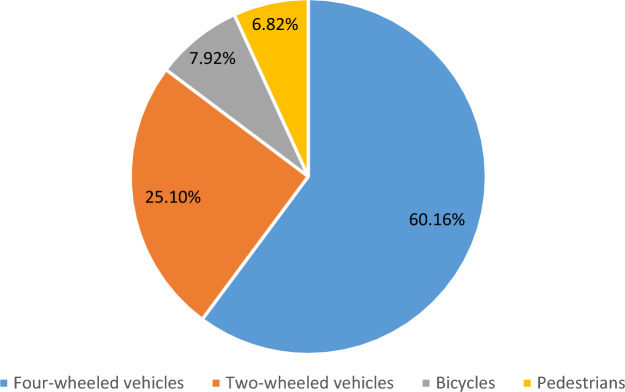
Details of the traffic accidents.

Concerning etiology, traffic accidents were the most common cause of traumatic SCI (n = 207/539, 38.40%), followed by falls (low falls 23.19%; n = 125/539, high falls 12.98%; n = 70/539), and machine-related injury (11.31%; n = 60). In addition, our results showed that four-wheeled vehicles were the leading cause of traffic accidents (62.16%), followed by two-wheeled vehicles (25.10%) Fig. 1. According to Table 2, the etiologies of injuries varied across different age groups. In the 30–39 age group, the most common causes of injury were traffic accidents, falls (both low and high falls), and machine-related injuries. Furthermore, most injuries occurred in the 30–39 age group, accounting for 39.70% of the total injuries (n = 214/539). The next highest proportion of injuries was observed in the 40–49 age group, representing 17.81% of the total injuries (n = 96/539). The mean age of each traumatic etiology was also distinct from each other. Notably, patients who were injured by low fall showed a significantly higher mean age of 51.5 ± 14.3 years compared to patients with other etiologies.

**Table 2:** Type and severity of injury, number of cases of each traumatic etiology according to age group.

Traumatic etiology	Traffic accidents	Low fall	High fall	Falling objects	Machinery- injury	Sport	Overall
Number of cases (%)	207(38.40%)	125(23.19%)	70(12.98%)	47(8.71%)	60(11.31%)	30(5.41%)	539(100%)
Mean age at injury	43.7 ± 12.4	51.5 ± 14.3	33.8 ± 12.2	36.5 ± 13.6	34.7 ± 14.2	27.1 ± 11.9	35.4 ± 13.2
$${\mathrm{Type and severity of injury}}^{{\text{a}}}$$							
Complete tetraplegia	35(6.49%)	4(0.74%)	24(4.45%)	9(1.66%)	10(1.85%)	2(0.37%)	84(15.58%)
Incomplete tetraplegia	54(10.01%)	13(2.41%)	32(5.93%)	16(2.96%)	19(3.52%)	5(0.92%)	139(25.78%)
Complete paraplegia	34(6.30%)	5(0.92%)	27(5.00%)	14(2.59%)	17(3.15%)	2(0.37%)	99(18.18%)
Incomplete paraplegia	75(13.91%)	31(5.75%)	56(10.38%)	22(4.08%)	25(4.63%)	8(1.48%)	217(40.46%)
$${\mathrm{Type and severity of injury}}^{{\text{b}} }({\text{yr}})$$							
0–19	12(2.22%)	6(1.11%)	2(0.37%)	1(0.18%)	1(0.18%)	7(1.29%)	29(5.38%)
20–29	30(5.56%)	24(4.45%)	12(2.22%)	6(1.11%)	16(2.96%)	5(0.92%)	93(17.25%)
30–39	81(15.02%)	39(7.23%)	32(5.93%)	26(4.82%)	24(4.45%)	12(2.22%)	214(39.70%)
40–49	36(6.67%)	28(5.19%)	15(2.78%)	6(1.11%)	11(2.04%)	0(0)	96(17.81%)
50–59	11(2.04%)	29(5.38%)	5(0.92%)	4(0.74%)	5(0.92%)	4(0.74%)	58(10.78%)
≥ 60	25(4.63%)	20(3.71%)	2(0.37%)	1(0.18%)	1(0.18%)	0(0)	49(9.08%)

Values are presented as number (%) or mean ± standard deviation.

^a^Numbers in parentheses indicate percentages among same etiology.

^b^Numbers in parentheses indicate percentages among same etiology and percentages among same age group.

Table 3 displays the mean age, AIS score, and the detailed number of cases of non-traumatic etiologies of spinal cord injury (SCI). The most prevalent cause of non-traumatic SCI was neoplasm, accounting for (34.54%; n = 38/110) of the cases. Among the non-traumatic cases, only six patients presented with complete injury (AIS-A), while the remaining 104 patients had incomplete injuries caused by non-traumatic factors.

**Table 3:** Number of cases, mean age and AIS of each non-traumatic etiology.

Non-traumatic etiology	Demyelinating disease	Neoplasm	Degenerative disease	Vascular disease	Infectious disease	Others	Overall
Number of cases (%)	21(19.09%)	38(34.54%)	11(10.02%)	14(12.72%)	19(17.27%)	7(6.36%)	110(100%)
Mean age at injury	55.3 ± 12.5	57.2 ± 10.4	43.8 ± 8.4	54.1 ± 14.1	51.9 ± 13.5	57 ± 12.4	54.8 ± 16.4
$${\mathrm{Type and severity of injury}}^{{\text{a}}}$$							
Complete tetraplegia	0(0%)	1(0.90%)	0(0%)	0(0%)	0(0%)	0(0%)	1(0.90%)
Incomplete tetraplegia	2(1,81%)	4(3.63%)	5(4.54%)	3(2.72%)	2(1.81%)	1(0.90%)	17(15.45%)
Complete paraplegia	0(0%)	1(0.90%)	1(0.90%)	1(0.90%)	0(0%)	0(0%)	3(2.72%)
Incomplete paraplegia	14(12.72%)	16(14.54%)	23(20.90%)	13(11.81%)	13(11.81%)	10(9.09%)	89(80.93%)
$${{\text{AIS}}}^{{\text{a}}}$$							
A	1(0.90%)	0(0%)	0(0%)	3(2.72%)	2(1.81%)	0(0%)	6(5.45%)
B	2(1.81%)	1(0.90%)	3(2.72%)	3(2.72%)	6(5.45%)	2(1.81%)	17(15.45%)
C	3(2.72%)	10(9.09%)	4(3.63%)	4(3.63%)	3(2.72%)	4(3.63%)	28(25.45%)
D	13(11.81%)	19(17.27%)	10(9.09%)	7(6.36%)	6(5.45%)	4(3.63%)	59(53.65%)

Values are presented as mean ± standard deviation or number (%).

AIS, American Spinal Injury Association impairment scale.

^a^Numbers in parentheses indicate percentages among same etiology.

### Type of disability and severity of injury

The types of disabilities and severity of injury resulting from spinal cord injury (SCI) are shown in Table 2. Among traumatic SCI patients, the majority (40.46%; n = 217/539) presented with incomplete paraplegia. In the case of traffic accidents and falls (low and high falls), most patients had incomplete paraplegia (13.91%; n = 75/593) and (16.13%; n = 87/539), respectively (Table 2). For non-traumatic causes of SCI, the most common outcome was incomplete paraplegia (89.93%; n = 89/110), followed by incomplete tetraplegia (15.45%; n = 17/110), Table 3.

### Treatment of TSCI and clinical complications disturbances of function among SCI individuals

Among all patients, a total of 57.31% (372/649) underwent surgical procedures such as laminoplasty, spinal decompression, fusion, and internal fixation. Additionally, the proportions of patients who received rehabilitation therapy, traditional therapy, and hyperbaric oxygen therapy were 64.08% (414/649), 58.04% (375/649), and 34.99% (229/649), respectively (Table 4).

**Table 4:** The treatment options for persons with SCI.

Treatment options	Traumatic	Non-traumatic	Overall
Surgery			
Yes	294 (54.55%)	78 (70.90%)	372(57.31%)
No	245(45.46%)	32 (29.09%)	277 (42.69%)
Rehabilitation therapy			
Yes	333 (61.78%)	81 (73.63%)	414 (64.08%)
No	206 (38.21%)	29 (26.36%)	235 (5.92%)
Traditional therapy			
Yes	301(55.84%)	74 (67.27%)	375 (58.04%)
No	238 (44.15%)	36 (32.72%)	274 (41.96%)
Hyperbaric oxygen therapy			
Yes	143 26.53%)	86 (78.18%)	229 (34.99%)
No	396 (73.46%)	24 (21.81%)	420 (65.01%)
Assistive devices			
Yes	275 (51.02%)	43 (39.09%)	318 (48.76%)
No	264 (48.97%)	67 (60.90%)	331 (51.24%)
Medicine			
Yes	93 (17.25%)	92 (83.63%)	185 (28.63%)
No	446 (82.93%)	18 (16.36%)	464 (71.37%)

Values are presented as number (%).

The hospitalization time for individuals with spinal cord injury (SCI) varied widely, ranging from 3 to 310 days. The average hospitalization duration was 24.31 days, with a standard deviation of 57.27. In the current study, 54.08% (351/649) of the SCI patients experienced clinical complications. The most common complications were urinary tract infection, affecting 17.10% (111/649) of the patients, followed by pulmonary infection at 15.65% (98/649), bedsores at 9.24% (60/649), hyponatremia at 6.92% (43/649), Table 5.

**Table 5:** The Clinical complication for persons with SCI.

Clinical complications	Traumatic	Non-traumatic	Overall
Urinary tract infection	93(17.25%)	18(16.36%)	111(17.10%)
Pulmonary infection	82 (15.21%)	16 (14.54%)	98 (15.65%)
Bedsores	49 (9.09%)	11 (10.0%)	60 (9.24%)
Hyponatremia	34(6.30%)	9(8.18%)	43(6.92%)
Deep venous thrombosis	16 (2.96%)	6 (5.45%)	22 (3.89%)
Other	12 (2.22%)	5 (4.54%)	17 (2.97%)

Values are presented as number (%).

The mean time of admission to the rehabilitation department was 41.7 (± 31.7) for TSCI patients, while it was 36.3 (± 20.3) for NTSCI patients, and this time was earlier among complete SCI patients compared with NTSCI. The Hospitalization time ranged between 3 and 291 days, with a mean length of 236.4 (± 63.8) days for TSCI patients and 86.8 (± 59.5) days for NTSCI, Table 6.

**Table 6:** Time of admission to the rehabilitation department and hospitalization time among patients with SCI.

	Traumatic SCI	Non-traumatic SCI
Time of admission to the rehabilitation department, d		
Complete injury	33.4 ± 42.1	21.8 ± 30.7
Incomplete injury	37.4 ± 51.2	27.2 ± 28.4
Total	41.7 ± 31.7	36.2 ± 20.3
Hospitalization time , d		
Complete injury	310.2 ± 157.1	178.7 ± 97.2
Incomplete injury	178.8 ± 97.6	73.1 ± 54.4
Total	236.4 ± 63.8	86.8 ± 59.5

Values are presented as mean ± standard deviations.

## Discussion

A recent systematic review of 17 studies conducted in China revealed that the epidemiological features of SCI differed across different regions^26,27^. This implies that tailored preventive measures must be implemented based on the specific characteristics of each region. This retrospective, cross-sectional study of SCI in Wuhan, Hubei, China, from 2016 to 2022 aimed to describe the demographic and clinical characteristics of patients with SCI. Since this study was conducted retrospectively, it was inevitable that some data might have been missing. However, efforts were made to minimize data loss by thoroughly examining all relevant medical records to ensure the resulting dataset was as comprehensive as possible.

The current findings showed that the male-to-female ratio of SCI patients was roughly 2.56:1, which is consistent with the findings of a study conducted in Wuhan^5^, which showed that 75.4% of patients with all-cause SCI were men, with a male-to-female ratio of 3:1

This sex imbalance was more profound in traumatic SCI patients, with a male-to-female ratio of 2.82:1 (398:141), which is consistent with or more significant than that in previous epidemiologic studies in China^20,24,25^. This tendency of dominance of male cases in traumatic SCI is also consistent with previous epidemiologic studies in other developed countries^28,29^. Meanwhile, non-traumatic SCI showed a smaller difference in the sex ratio at 1.68:1 (69 males, 41 females) in our study, which is closely consistent (1.47:1) with a recent study by Shin et al.^30^.

The most prevalent occupational groups in this study were workers (28.20%) and peasants (17.02%). This outcome is consistent with previous results from Tianjin and Chongqing^5,22,25^. This finding was primarily due to these patients’ low educational background, which restricted them to manual labor and raised their susceptibility to SCI, which may explain the higher burden of TSCI in this occupational category. Our findings showed that the proportion of married patients is higher than that of unmarried patients, which could be because most patients were middle-aged when most Chinese people marry.

With respect to the etiology of traumatic SCI, this study found that the primary causes of TSCI in Wuhan were traffic accidents (38.40%), falls (low and high falls) (36.17%), machine-related injury (11.31%), falling objects (8.71%), and sport-related injury (5.41%). These findings contradict previous studies, which revealed that falls (52.3%) were the primary cause, followed by motor vehicle accidents (36.4%)^21,22,25^.

Traditionally, traffic accidents have been the main cause of SCI in most developed countries, and the majority of high falls resulting in TSCI happened in the construction industry^31–33^. This can be attributed to China’s quickening industrialization process and its growing massive infrastructure projects. Therefore, some preventive public health strategies should be taken, such as using seatbelts, and environmental adjustments should be reinforced to reduce the occurrence of SCI; this can include expanding and improving road infrastructure and installing protective barriers between motorways and sidewalks to minimize the risk of traffic accidents^34^. Additionally, it is necessary to develop significant fall prevention measures in the construction industry.

High-energy injuries, such as those caused by traffic accidents and falling objects, were the leading causes of TSCI in young individuals. In contrast, low-energy injuries, such as those caused by low falls, were more common in the elderly^35^. SCIs are caused by various factors, including falls (both high and low), traffic accidents, impact with falling objects, sports, and violent injuries, and these factors differ by country and region. In 2006, an epidemiological survey in Canada revealed that traffic accidents were the leading cause of SCIs, while falls (both high and low) became the leading cause in 2009^35,36^. Another survey study from seven Middle Eastern and North African (MENA) countries showed that traffic accidents are still the primary cause of SCIs, followed by falls (both high and low), violence, and sports^37^.

As this study showed that four-wheeled vehicles were the leading cause of traffic accidents (62.16%), followed by two-wheeled vehicles (25.10%). This outcome is consistent with the survey study conducted in Japan among TSCI patients, which showed that four-wheeled vehicles were responsible for (46.32%) of traffic accidents, while two-wheeled vehicles were responsible for (26.65%)^38^. Cervical injuries were the most frequent level of injury identified in the current study, accounting for 41.9% of all cases. Previous research also showed that 55% to 75% of all spinal cord injuries were cervical injuries, which were the most frequent level^36,37^.These findings could be explained by the cervical vertebrae’s comparatively low mechanical stability, which makes them more prone to trauma than any other part of the vertebral column. Among non-traumatic etiologies, neoplasm was the single most common cause of NTSCI, accounting for 34.54%, followed by demyelinating disease (19.09) and infectious disease (17.27) as the second most common causes. Vascular disease accounted for (12.71%) and degenerative disease followed at (10.0%).

Concerning disability types and severity of injury, the epidemiologic study by Shin et al.^30^ in 2013 showed incomplete tetraplegia as the most common type, accounting for 36.9% of all SCIs. According to annual reports of NSCISC^39^ and as Jain et al.^40^ suggest, the proportion of cervical spinal cord injury and incomplete injury has increased gradually in the United States. In our study, 241 (37.42%) patients had tetraplegia and only 85 (13.09%) of them presented complete injury. A notably low proportion of complete injury might be attributed to advances in emergency medical care, trauma management, and transportation systems, which have significantly improved over the years and prompt and effective medical intervention following the injury and early admission to the spinal care units; early access to specialized care after acute TSCI is associated with improved outcomes^41,42^. Patients who are admitted to specialist spinal care units within 24 h of injury had fewer secondary complications^41,43^. Early admissions into a specialized spinal care unit have also been found to lead to decreased in-hospital rehabilitation time compared with initial admission to trauma care without specialist SCI expertise^43^.

It is well known that patients with SCI experience many complications. Based on the current findings, 51.3% of SCI patients developed clinical complications, and the four most frequent complications were urinary tract infections (17.10%), pulmonary infections (15.65%), bedsores (9.24%), and hyponatremia (6.92%). These findings are congruent with a previous study performed in northwest China. That study revealed that (36.4%) of people with SCI experienced one or more complications, and the most frequent complications were pulmonary infections (32.5%), followed by hyponatremia (24.1%), bedsores (16.3%), and urinary tract infections (12.5%)^14^. In addition, another study conducted in Italy showed that the four most frequent complications of SCI were pain, urinary tract infections, lung infections, and bedsores^44^. Urinary tract infections and pulmonary infections were considered the most common complications among people with SCI, and they were primarily attributed to inadequate nursing practices in hospitals^44^. Respiratory problems are associated with long-term bed rest, rib fractures, and smoking-related respiratory disease. In addition, SCIs at the cervical level can impair diaphragm or intercostal muscle function, impede respiration, and rise to cough, making sputum removal difficult. Such symptoms may also appear as consequences of respiratory disease^45^. The risk of pulmonary infection increases with a high level of SCI injury. SCI above the C5 level leads to diaphragm dysfunction and the risk of a pulmonary infection can increase up to 90%^46^. These findings highlight the need to provide adequate care to patients to reduce hospitalization time and improve prognosis.

Based on the evaluation and analysis of the epidemiological features of spinal cord injury (SCI) in Hubei province, China, several recommendations and interventions can be proposed. First and foremost, there is a clear need for further research on the epidemiology of SCI in this specific region to gain a comprehensive understanding of the scope and characteristics of the problem. Preventive efforts should be targeted toward individuals who are most vulnerable to SCI, such as young male adults engaged in hazardous outdoor occupations. These can involve implementing specific safety measures, providing proper training and protective equipment, and promoting awareness of potential risks and preventive measures. Environmental adjustments should be reinforced to reduce the occurrence of SCI^34^. Education plays a crucial role in preventing SCI^47^. Educational courses can be tailored to specific demographic groups based on age, gender, and occupation. These courses can focus on raising awareness about SCI, promoting safe practices, and emphasizing the importance of using protective instruments and helmets, particularly in high-risk occupations. Legislation should also be considered to enforce protective equipment use and penalize individuals who violate road laws, thus ensuring compliance and accountability^30^. Lastly, recognizing the significance of SCI physiotherapy and rehabilitation is vital.

A better understanding of the epidemiology of SCI can inform future research by identifying risk factors, unraveling causal pathways, tailoring prevention strategies, evaluating interventions, and tracking trends. By incorporating epidemiological knowledge into research efforts that help to make significant strides in preventing and mitigating the impact of SCI.

## Limitations

Several limitations in this study have to be considered. First, the current study was a hospital-based descriptive study that determined only a small proportion of all people with SCI; furthermore, Hubei Province does not currently have an SCI-registered system; thus, the exact incidence rate could not be established. Second, the data are collected retrospectively. Consequently, some data loss was unavoidable. Third, we missed some data since many complications and treatments were not adequately diagnosed or documented in medical records. Fourth, people who died at the scene of the accident or on the way to the hospital were not included, which may have resulted in an underestimation of the prevalence rate. Lastly, we could only check the patients’ AIS upon admission or when they were transferred to our department. Therefore, the timing of evaluation of severity and type of SCI varied, and this could have influenced the severity of the injury itself. Despite these limitations, our study remains valuable because, to our best knowledge, this is the latest study that reports the epidemiology and etiology of SCI in China.

## Conclusion

In conclusion, this study demonstrates the cross-sectional analysis of the clinicodemographic characteristics of SCI patients in Wuhan’s Tongji Hospital. The findings of our study indicated a higher proportion of males, with falls and traffic accidents being the top two causes of injury among TSCI patients. Farmers and workers were identified as the occupations most susceptible to SCI. It is crucial to prioritize the risk of falling among older adults and allocate more attention to this issue. These results emphasize the need for tailored preventive strategies that consider the unique characteristics of different types of SCI patients, Lastly, the significance of SCI rehabilitation should be underscored.

## Data Availability

The data generated in this study are available from the corresponding author on reasonable request.
